# Monthly Record of Deaths in the City of Buffalo

**Published:** 1857-12

**Authors:** C. L. Dayton

**Affiliations:** Health Physician


ART. V.— *Report of Mortality in Buffalo for the month of Oct.,* 1857.

By C. L. DAYTON, M. D., Health Physician.

| DISEASES. | No. | Males. | Females. |
|---|---|---|---|
| Accident, | 8 | 7 | 1 |
| Apoplexy, | 1 | | 1 |
| Asphyxia Immersorum, | 3 | 3 | |
| Bronchitis, | 1 | 1 | |
| Croup, | 1 | 1 | |
| Cholera Infantum, | 15 | 7 | 7 |
| Cephalitis, | 3 | 3 | |
| Diarrhœa, | 13 | 2 | 11 |
| Dentition, | 6 | 2 | 3 |
| Dysentery | 14 | 10 | 4 |
| Debilitas Senilis, | 7 | 4 | 3 |
| Eclampsia, | 20 | 10 | 10 |
| Febris Typhoid, | 6 | 2 | 4 |
| " Scarlatina, | 5 | 2 | 3 |
| " Remittent, | 2 | 1 | 1 |
| " Ignotus, | 1 | 1 | |
| Hernia, | 1 | | 1 |
| Hyperæmia Cerebralis, | 1 | | 1 |
| " Pulmonalis, | 1 | | 1 |
| Hydrocephalus, | 4 | 2 | 2 |
| Ignotus, | 26 | 15 | 11 |
| Laryngitis, | 1 | | 1 |
| Marasmus, | 7 | 4 | 3 |
| Morbus Cordis, | 2 | 2 | |
| " Cerebralis, | 1 | 1 | |
| Paralysis, | 2 | | 2 |
| Pertussis, | 7 | 5 | 2 |
| Pneumonitis, | 4 | 1 | 3 |
| Rubeola, | 3 | 2 | 1 |
| Syphilis, | 1 | | 1 |
| Still-born, | 12 | 9 | 2 |
| Tumor Ovarian, | 1 | | 1 |
| Tabes Mesenterica, | 3 | 2 | |
| Tuberculosis Pulmonalis, | 19 | 9 | 10 |
| Total, | 202 | | |

SEXES.

| | |
|---|---|
| Males, | 108 |
| Females, | 82 |
| Sex not given, | 12 |
| Total, | 202 |

AGES.

| | | | |
|---|---|---|---|
| Still-born, | 13 | Between 20 years and 30 years, | 12 |
| 1 day, | 6 | " 30 " " 40 " | 9 |
| 1 day and 30 days, | 11 | " 40 " " 50 " | 10 |
| Between 1 month and 6 months, | 30 | " 50 " " 60 " | 14 |
| " 6 months and 12 " | 13 | " 60 " " 70 " | 10 |
| " 1 year " 3 years, | 20 | " 70 " " 80 " | 3 |
| " 3 " " 5 " | 16 | " 80 " " 90 " | 1 |
| " 5 " " 10 " | 7 | " 90 " " 100 " | 0 |
| " 10 " " 20 " | 7 | " 100 " | 0 |
| Total number under 10 years, | 116 | Total number over 10 years, | 66 |
| Ages not given, | 2 | Total, | 202 |

NATIVITIES.

| | | | |
|---|---|---|---|
| American, (white,) | 109 | Prussian, | 0 |
| " (colored,) | 3 | Saxon, | 0 |
| German, | 41 | French, | 1 |
| Irish, | 15 | Scotch, | 0 |
| English, | 5 | Poland, | 0 |
| Canadian, | 1 | Country not given, | 27 |
| Total, | | | 202 |

*Electricity in the Suppression of the Lacteal Secretion.*—M. Becquerel, in a late communication to the Société Médicale des Hôpitaux de Paris, has made some remarks upon the influences of electricity in restoring the secretion of milk. His attention was called to the subject by a case related to him by M. Aubert, who had employed electricity in the case of a young woman, whose milk had been suppressed in consequence of a double pneumonia. The electricity was applied to the breast by means of moist excitors, and after four applications, each lasting twenty minutes, the lacteal secretion was completely restored.—*British and Foreign Medico-Chirurgical Review*, from *Cincinnati Med. Observer.*

*Extraordinary Fecundity.*—It is stated in a recent No. of the Magazine of Natural History, &c., of Moscow, that the peasant Kirilow was presented, along with his wife, to the Empress. This peasant was married, for the second time, at the age of 70. His first wife was delivered twenty-one times: four times of four infants at a birth; seven times of three infants, and ten times of twins: in all, fifty-seven children then alive. The second wife nad already been delivered seven times: once of triplets; and six times of twins: in all, fifteen living children.—*Med. News.*

